# Cardiovascular effects of metyrapone treatment in patients with mild autonomous cortisol secretion: a secondary analysis

**DOI:** 10.1210/clinem/dgag228

**Published:** 2026-06-11

**Authors:** Helena Niziolek, Ivica Just, Clemens Baumgartner, Anna Tosin, Hansjörg Habisch, Paul Fellinger, Hannes Beiglböck, Michael Poledniczek, Luise Bellach, Anton Luger, Alexandra Kautzky-Willer, Siegfried Trattnig, Maximilian Zeyda, Thomas Scherer, Marie Helene Schernthaner-Reiter, Florian W Kiefer, Greisa Vila, Andreas Kammerlander, Tobias Madl, Michael Leutner, Martin Krssak, Michael Krebs, Peter Wolf

**Affiliations:** Division of Endocrinology and Metabolism, Department of Internal Medicine III, Medical University of Vienna, Vienna 1090, Austria; Division of Endocrinology and Metabolism, Department of Internal Medicine III, Medical University of Vienna, Vienna 1090, Austria; Division of Endocrinology and Metabolism, Department of Internal Medicine III, Medical University of Vienna, Vienna 1090, Austria; Division of Endocrinology and Metabolism, Department of Internal Medicine III, Medical University of Vienna, Vienna 1090, Austria; Division of Medicinal Chemistry, Otto Loewi Research Center for Vascular Biology, Immunology and Inflammation, Medical University of Graz, Graz 8010, Austria; Division of Endocrinology and Metabolism, Department of Internal Medicine III, Medical University of Vienna, Vienna 1090, Austria; Division of Endocrinology and Metabolism, Department of Internal Medicine III, Medical University of Vienna, Vienna 1090, Austria; Division of Cardiology, Department of Internal Medicine II, Medical University of Vienna, Vienna 1090, Austria; Division of Endocrinology and Metabolism, Department of Internal Medicine III, Medical University of Vienna, Vienna 1090, Austria; Division of Endocrinology and Metabolism, Department of Internal Medicine III, Medical University of Vienna, Vienna 1090, Austria; Division of Endocrinology and Metabolism, Department of Internal Medicine III, Medical University of Vienna, Vienna 1090, Austria; Department of Biomedical Imaging and Image Guided Therapy, Medical University of Vienna, Vienna 1090, Austria; Division of Pediatric Pulmonology, Allergology and Endocrinology, Department of Pediatrics and Adolescent Medicine, Medical University of Vienna, Vienna 1090, Austria; Division of Endocrinology and Metabolism, Department of Internal Medicine III, Medical University of Vienna, Vienna 1090, Austria; Division of Endocrinology and Metabolism, Department of Internal Medicine III, Medical University of Vienna, Vienna 1090, Austria; Division of Endocrinology and Metabolism, Department of Internal Medicine III, Medical University of Vienna, Vienna 1090, Austria; Division of Endocrinology and Metabolism, Department of Internal Medicine III, Medical University of Vienna, Vienna 1090, Austria; Division of Cardiology, Department of Internal Medicine II, Medical University of Vienna, Vienna 1090, Austria; Division of Medicinal Chemistry, Otto Loewi Research Center for Vascular Biology, Immunology and Inflammation, Medical University of Graz, Graz 8010, Austria; BioTechMed-Graz, Graz 8010, Austria; Division of Endocrinology and Metabolism, Department of Internal Medicine III, Medical University of Vienna, Vienna 1090, Austria; Division of Endocrinology and Metabolism, Department of Internal Medicine III, Medical University of Vienna, Vienna 1090, Austria; Division of Endocrinology and Metabolism, Department of Internal Medicine III, Medical University of Vienna, Vienna 1090, Austria; Division of Endocrinology and Metabolism, Department of Internal Medicine III, Medical University of Vienna, Vienna 1090, Austria

**Keywords:** mild autonomous cortisol secretion, adrenal adenoma, chronotherapy, cardiovascular risk profile

## Abstract

**Context:**

Mild autonomous cortisol secretion (MACS) is associated with increased mortality, mainly because of cardiovascular disease.

**Objective:**

Here we investigated the effects of cortisol-lowering medical treatment on blood pressure regulation, cardiac fat depots, heart function and morphology, and other cardiovascular risk factors.

**Methods:**

In this secondary analysis of our prospective, open-label, single-center study at the Medical University of Vienna, we investigated 15 patients with MACS (12 women; age 59 [53-64] years) before and after 12 weeks of treatment with evening doses of metyrapone (500 mg at 6 Pm and 250 mg at 10 Pm). Cardiac fat stores, morphology, and myocardial function were evaluated using cardiac magnetic resonance imaging and spectroscopy. Orthostatic blood pressure regulation was measured by standardized protocols. Lipidomics, renin-angiotensin-aldosterone-system activity and branched-chain amino acid (BCAA) concentrations were assessed.

**Results:**

Mean supine systolic (137 [121-139] vs 122 [115-129] mmHg; *P* = 0.041) and diastolic (89 [80-93] vs 75 [71-86] mmHg; *P* = .045) blood pressure decreased after treatment in patients without changes in concomitant antihypertensive medication. Epicardial fat was lower at follow-up compared to baseline (1032.70 [894.23-1482.90] vs 929.37 [736.12-1317.15] mm^2^; *P* = .022). No changes in paracardial and intramyocardial fat, as well as in cardiac function and morphology were observed. The aldosterone-to-angiotensin II ratio was lower at follow-up (1.56 [0.87-2.82] vs 1.21 [0.98-1.92]; *P* = .042) and alterations in lipid profiles, but not in BCAA levels, were identified.

**Conclusion:**

Treatment with evening doses of metyrapone improved blood pressure and reduced epicardial adipose tissue.

Mild autonomous cortisol secretion (MACS) is the most common hormonal abnormality in patients with adrenal adenomas, diagnosed in about 20% to 50% of patients. MACS is associated with numerous cardiovascular comorbidities and an increased mortality, especially in women younger than 65 years (1).

With regard to “classic” cardiovascular risk factors, the prevalence of hypertension, dyslipidemia, central obesity, and diabetes mellitus is increased in patients with MACS (2). Additionally, higher levels of branched-chain amino acids (BCAAs), an established biomarker for adverse cardiometabolic disorders, have been described (3). This translates into a higher risk for cardiovascular events (4).

Hypercortisolism affects cardiac structure and function in patients with overt Cushing syndrome (CS) (5), but also in patients with MACS cardiac remodeling is more pronounced compared to control populations (6). Importantly, changes in cardiac fat depots might additionally contribute to structural and functional alterations of the heart. Here, epicardial adipose tissue (EAT) might be of particular interest, as it is strongly associated with the presence of hypercortisolism in patients with overt CS (7, 8), as well as in patients with adrenal adenoma and MACS (9). Being located between the myocardium and the visceral layer of the pericardium, EAT directly surrounds the coronary arteries and exerts paracrine actions on the myocardium that promote myocardial fibrosis, endothelial and contractile dysfunction, and atherosclerosis (10-12). Moreover, increased intramyocardial lipid accumulation probably linked with visceral obesity, dyslipidemia, and insulin resistance might exert additional lipotoxic injury on cardiomyocytes (13).

Despite its substantial clinical effect, therapeutic management of MACS is not well defined. Recently published prospective studies report beneficial effects on blood pressure (14), body weight, and glucose metabolism following adrenalectomy in patients with MACS (15). However, evidence on the effects of pharmacological treatment options is scarce. We recently reported significant improvements in hepatic lipid content, insulin sensitivity, and systemic inflammation following short-term chronotherapy with evening doses of metyrapone, a selective 11β-hydroxylase inhibitor, in patients with MACS (16).

The aim of this substudy was to investigate predefined secondary cardiovascular endpoints, focusing on cardiac function, morphology, and fat stores. In addition, the effect of therapy on orthostatic blood pressure regulation, the lipid profile, and circulating concentrations of BCAAs was assessed.

## Material and methods

This study is a secondary analysis of a prospective, exploratory, open-label, single-center study (AdrenalClock Study) conducted at the Division of Endocrinology and Metabolism at the Medical University of Vienna. The study was conducted according to the guidelines of the Declaration of Helsinki and was approved by the local ethics committee (1062/2022) and registered at EudraCT (2022-000161-40). The aim of the study is investigating effects metyrapone treatment for 12 weeks on cardiovascular risk profile in patients with MACS.

### Participants

Eligibility criteria and recruitment strategies were previously published by our study group (16). Adult patients with unilateral or bilateral adrenal adenoma(s) confirmed by computed tomography scan or magnetic resonance imaging (MRI) and the diagnosis of MACS were consecutively enrolled. MACS was defined by a morning cortisol level greater than 1.8 µg/dL without overt clinical signs of CS according to recent guidelines (17). Exclusion criteria encompassed insulin therapy, glycated hemoglobin A_1c_ greater than 8%, treatment with more than 4 antihypertensive agents, chronic kidney disease (estimated glomerular filtration rate < 45), liver disease (alanine transaminase/aspartate transaminase > 3× upper limit of normal), breastfeeding, pregnancy, adrenal adenomas with malignancy criteria, concomitant therapy with strong modulators of cytochrome P450 3A4 (CYP3A4) and glucocorticoid therapy within 3 months before study initiation, as well as general contraindications for MRI measurements. Written informed consent was obtained from all study participants prior to study inclusion.

### Procedures and outcomes

Patients were investigated before and after 12 weeks of treatment with evening doses of metyrapone (Metycor). Metyrapone was administered with a dose of 500 mg at 6 Pm and 250 mg at 10 Pm, analogous to the protocol published by Debono et al (18). At study initiation, patients were carefully educated regarding potential side effects of medication, handling on sick days, and course of action in case of signs of adrenal insufficiency. Hydrocortisone was provided as rescue medication.

Parameters of hypothalamus-pituitary-adrenal axis activity—serum cortisol, adrenocorticotropin (ACTH), dehydroepiandrosterone sulfate (DHEAS), and 11-deoxycortisol (11-DOC)—and electrolytes were measured at baseline and follow-up. Urinary free cortisol and midnight free salivary cortisol were self-collected and brought to the study visits before and after treatment. Sample analysis was conducted by routine laboratory methods at Medical University of Vienna using immunoassays (https://labormedizin.meduniwien.ac.at).

The following parameters were assessed at study initiation and after 12 weeks of treatment:

### Cardiac function and morphology

All patients underwent cardiac MR using a whole-body high-field 3 Tesla Prisma Fit scanner (Siemens Healthineers) equipped with a spine coil and a flexible body coil from the same manufacturer. A supine measuring position was maintained by patients, and image recording was conducted while end-expiratory breath held.

Measurement of cardiac function was performed with electrocardiogram–gated cine true fast imaging with steady-state precession (TrueFISP) sequences. Acquisition of images was conducted in standard, 2-chamber, 4-chamber, and short-axis, exhibiting 10 to 12 equidistant slices spanning from the apex to the base of the left ventricle. Determination of cardiac output (CO), stroke volume (SV), ejection fraction (EF), peak ejection rates, filling rates, left ventricular myocardial mass, end systolic volume (ESV), and end diastolic volume (EDV) were conducted. All listed parameters were gained using short axis images over the whole cardiac phases via syngo.via software (Siemens Healthineers) by semiautomated contouring of endocardial and epicardial borders. Parameters were presented with standard units and normalized or adjusted to body surface area (BSA).

### Epicardial and paracardial adipose tissue

The MRI 4-chamber short-axis view of the heart was depicted. Three different time points were visually assigned for analysis according to the cardiac cycle phases. The end diastolic phase was mapped as the largest ventricle expansion of the cine-MRI stack, the systolic phase as the minimum ventricle expansion, and the third time point was chosen as the midpoint value between the aforementioned slices. EAT is defined as adipose tissue surrounding the myocardium, bordered by the visceral layer of the pericardium. The paracardial adipose tissue is located outside the parietal layer of the pericardium in the mediastinum (19) (Fig. 1). The borders between epicardial and paracardial tissue were visually assessed, and the areas were manually followed and marked using FIJI software (20). Paracardial adipose tissue was measured only as connected tissue directly surrounding the pericardium. Subsequently, the mean values for the 3 epicardial and paracardial areas were calculated.

**Figure 1:**
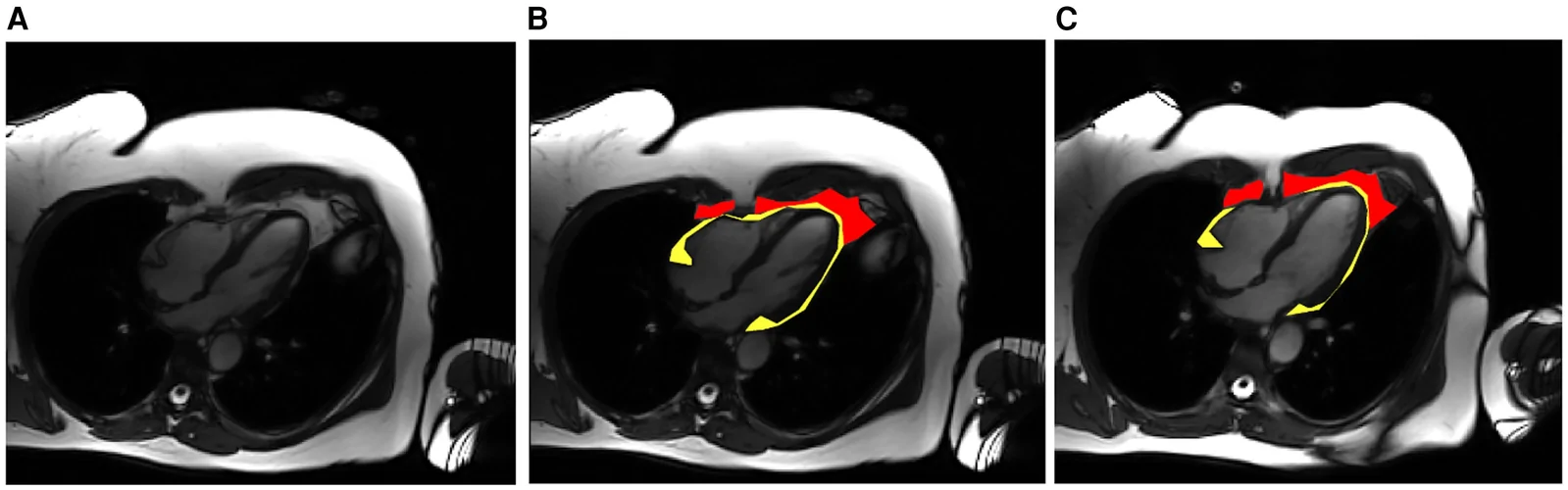
Mapping of epicardial adipose tissue using FIJI software and magnetic resonance imaging (MRI). MRI panels shown from left to right: A, MRI at baseline; B, mapping of epicardial (yellow) and paracardial adipose tissue (red) at baseline; C, FIJI mapping after treatment. A significant decline of epicardial adipose tissue (yellow) was observed after 12 weeks of metyrapone treatment (*P* = .022), while paracardial adipose tissue remained unchanged (red).

### Intramyocardial lipid content

Assessment of intramyocardial lipid content was performed using electrocardiogram–gated proton magnetic resonance spectroscopy (^1^H-MRS). For confounder reduction through epicardial fat, a volume of interest was positioned at the interventricular septum. PRESS sequences were applied for positioning and signal reception during patients’ single-breath hold. Spectral data analysis was conducted using the spectroscopy analysis software by the manufacturer (Siemens Healthineers). The amount of intramyocardial lipid content was calculated as the ratio of the combined signal intensities of the methylene and methyl resonances to the water signal and expressed as a percentage (fat fraction) to total signal. Corrections for T_1_ and T_2_ relaxation were performed with reference values assessed at 3 Tesla in the skeletal muscle (21).

### Orthostatic blood pressure regulation

Orthostatic regulation of blood pressure and heart rate (Schellong test) was assessed by the same blood pressure device in supine and standing position 5 times in a row for 10 minutes, and both mean and singular values were used for subsequent analysis.

### Renin-angiotensin-aldosterone system

Equilibrium levels of angiotensin I, angiotensin II, and aldosterone were measured following ex vivo equilibration and subsequent stabilization of conditioned plasma samples as described previously (22, 23). Samples were spiked with stable isotope-labeled internal standards for individual angiotensins (200 pg) and deuterated (D4) aldosterone (500 pg). Following C18-based solid-phase-extraction, samples were subjected to liquid chromatography–tandem mass spectrometry analysis using a reversed-phase analytical column (Acquity UPLC C18, Waters) operating in line with a XEVO TQ-S triple quadrupole mass spectrometer (Waters Xevo TQ/S) in multiple reaction monitoring mode. Internal standards were used to correct for analyte recovery across the sample preparation procedure in each individual sample. Analyte concentrations were calculated from integrated chromatograms considering the corresponding response factors determined in appropriate calibration curves in serum matrix, on condition that integrated signals exceeded a signal-to-noise ratio of 10. Equilibrium levels of angiotensin I, angiotensin II, and aldosterone levels were used in renin-angiotensin-aldosterone system (RAAS)-Triple-A evaluation, involving the calculation of angiotensin-based markers for plasma-renin-activity (PRA-S: angiotensin I + angiotensin II), angiotensin-converting-enzyme (ACE-S: angiotensin II/angiotensin I), and the AA2 ratio (aldosterone/angiotensin II), as marker for adrenal responsiveness to angiotensin II signaling in releasing aldosterone.

### Lipidomics

Serum samples were directly quantified using a Bruker 600 MHz Avance Neo nuclear magnetic resonance (NMR) spectrometer (Bruker) as previously published (24). After thawing, 300 µL of serum were mixed 1:1 300 µL with Bruker In Vitro Diagnostics research buffer and analyzed according to the In Vitro Diagnostics workflow; Spectra were processed in TopSpin 4.4 and quantified via the Bruker Online Server using B.I.LISA for lipoprotein subclass analysis and B.I.Quant-PS for small-molecular-weight metabolites algorithms.

### Branched-chain amino acids

Concentrations of the analytes valine, isoleucine, leucine, and L-alloisoleucine were determined with a Shimadzu 8050 LCMS using MassChrom Amino Acid Kit (Chromsystems) (25). The single values of quantified BCAA concentrations as well as the total sum of valine, leucine, and isoleucine were calculated.

### Sample size calculation and statistical analysis

This is an exploratory analysis of prespecified secondary outcome parameters. No sample size calculation was performed for the included parameters. Continuous outcome variables were presented as medians (Q1-Q3), and qualitative variables were reported as absolute numbers (percentages). The Spearman correlation coefficient was used for the assessment of all continuous variables due to the limited sample size. The Wilcoxon signed-rank test was applied for comparing cardiometabolic outcomes between baseline and follow-up. Given the exploratory nature of our study, no corrections for multiple testing were performed. Two-sided *P* values less than .05 were considered statistically significant. R (version 4.4.1 [2024-06-14]) and GraphPad Prism Version 10 were used for analysis.

## Results

### Study population

Details on the study cohort, anthropometric characteristics, and tolerability were previously reported (16) and are summarized in Table 1. Fifteen patients (12 women) were investigated at baseline and 12 weeks after treatment with evening doses of metyrapone and completed all study procedures. Twelve patients with hypertension and concomitant intake of antihypertensive medication were enrolled. Information on demographic and biochemical details, as well as individual antihypertensive and lipid-lowering medication at baseline and follow-up, is reported in Supplementary Table S1 (26).

**Table 1:** Baseline patient characteristics (median; Q1-Q3)

Baseline characteristics	
No. of patients	15
Age (Q1-Q3), y	59 (53-64)
Sex (women in %)	12 (80)
Unilateral disease, n (%)	8 (53)
Body weight in kg (Q1-Q3)	75.20 (69.80-85.00)
Height in cm (Q1-Q3)	162 (158-169)
BMI (Q1-Q3)	28 (25-32)
Presence of arterial hypertension, n (%)	12 (80)
Cortisol after DST in µg/dL (Q1-Q3)	2.90 (2.35-4.60)
Morning serum cortisol, µg/dL (Q1-Q3)	11.70 (9.98-14.38)
ACTH, pg/mL (Q1-Q3)	4 (4-6)
DHEAS, µg/mL (Q1-Q3)	0.46 (0.26-0.83)
Mean*^a^* late-night saliva cortisol, µg/dL (Q1-Q3)	0.07 (0.05-0.08)
Mean*^a^* 24-h urinary free cortisol, µg/24 h (Q1-Q3)	74.72 (57.71-123.95)

Data were previously published and adapted (16).

Abbreviations: ACTH, adrenocorticotropin; BMI, body mass index; DHEAS, dehydroepiandrosterone sulfate; DST, dexamethasone suppression test.

^a^Mean values for 24-h urinary free cortisol and midnight saliva cortisol were calculated from 2 samples per patient. In case of only one available sample, the single value was used.

### Orthostatic blood pressure regulation

After 12 weeks of metyrapone treatment, mean systolic and diastolic supine values decreased nonsignificantly during orthostatic testing in the whole cohort. After excluding 4 patients with dose reductions in concomitant antihypertensive therapy from analysis, the decline of both systolic and diastolic supine mean values was statistically significant, while no further alterations in orthostatic regulation were observed (Table 2, Fig. 2). Statistical analysis was cross-checked independently to ensure reproducibility. Details on changes of concomitant antihypertensive treatment are reported in Supplementary Table S1 (26).

**Figure 2:**
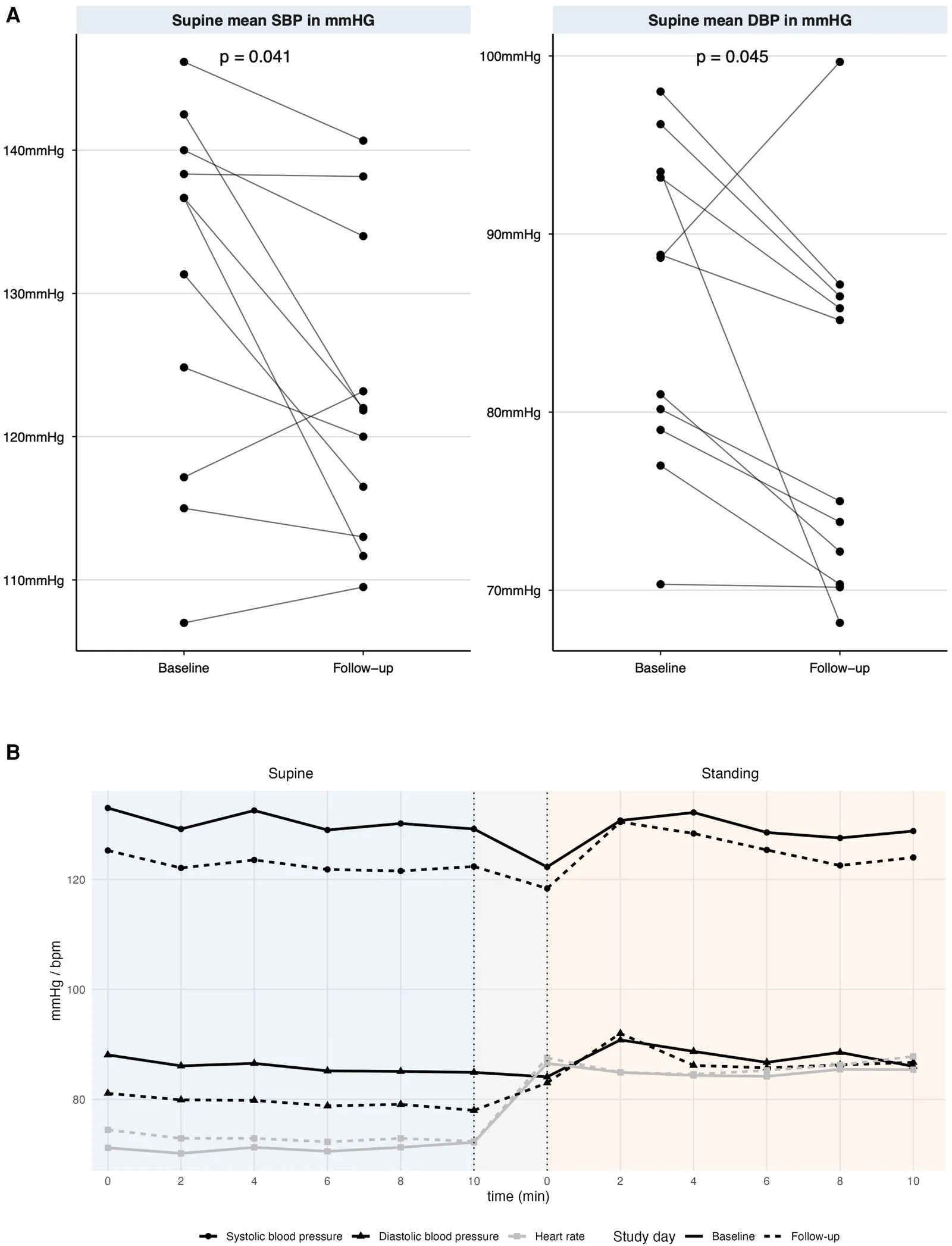
A, Supine mean systolic blood pressure (SBP) and supine mean diastolic blood pressure (DBP) were compared at baseline and follow-up in the subcohort of patients without any changes in antihypertensive treatment during the study period. B, Orthostatic blood pressure regulation testing with SBP and DBP and heart rate in supine and standing position at baseline (solid line) and follow-up (dotted line).

**Table 2:** Descriptive statistics (median; Q1-Q3) of blood pressure measurements in standardized orthostatic testing; the subcohort includes 11 patients, as 4 patients, who reduced antihypertensive medication due to low blood pressure during study period, were excluded

	Before treatment	After treatment	*P*
Whole cohort (n = 15)			
SBP supine mean, mm Hg	125 (116-138)	122 (112-125)	.078
SBP standing mean, mm Hg	124 (116-133)	127 (118-135)	.842
DBP supine mean, mm Hg	80 (76-91)	75 (71-86)	.105
DBP standing mean, mm Hg	86 (80-89)	83 (77-90)	.561
Heart rate supine mean, bpm	69 (64-78)	71 (67-72)	.167
Heart rate standing mean, bpm	85 (74-91)	85 (81 –87)	.720
Subcohort without changes in antihypertensive treatment (n = 11)			
SBP supine mean, mm Hg	137 (121-139)	122 (115-129)	.041
SBP standing mean, mm Hg	125 (118-139)	127 (116-135)	.320
DBP supine mean, mm Hg	89 (80-93)	75 (71-86)	.045
DBP standing mean, mm Hg	88( 81-89)	89 (81-98)	.831
Heart rate supine mean, bpm	69 (67-74)	71 (70-72)	.148
Heart rate standing mean, bpm	87 (78-90)	85 (82-87)	.831

Abbreviations: bpm, beats per minute; DBP, diastolic blood pressure; SBP, systolic blood pressure.

Baseline systolic (SBP; *r* = 0.574; *P* = .025) and diastolic (DBP; *r* = 0.568; *P* = .027) blood pressure positively correlated with left ventricle end-diastolic myocardial mass. No association between blood pressure and concentrations of serum cortisol, ACTH, 24-hour urinary cortisol excretion, or late-night saliva cortisol was observed. All parameters of orthostatic blood pressure regulation in the whole cohort and in the subcohort are reported in detail in Table 2.

### Cardiac fat stores, morphology, and function

EAT significantly declined after metyrapone treatment. Intramyocardial lipid content and paracardial adipose tissue remained unchanged after the treatment period (Table 3).

**Table 3:** Descriptive statistics (median; Q1-Q3) of cardiac morphology and function, as well as cardiac adipose tissue depots at baseline and at follow-up study visit

	Before treatment	After treatment	*P*
Cardiac morphology and function parameters (n = 15)			
EF, %	62.07 (57.64 to 65.39)	60.79 (57.20 to 63.87)	.488
HR	63 (60 to 69)	63 (60.5 to 68.5)	.916
EDV, mL/m^2^	64.16 (62.20 to 74.25)	65.43 (59.26 to 76.53)	.229
ESV, mL/m^2^	26.09 (23.61 to 29.39)	28.41 (21.86 to 29.63)	.804
Stroke volume, mL/m^2^	40.13 (37.39 to 46.64)	38.65 (37.03 to 43.48)	.525
Cardiac index, L/min/m^2^	2.74 (2.48 to 3.00)	2.51 (2.33 to 2.98)	.842
End-diastolic left ventricular myocardial mass, g/m^2^	63.74 (50.26 to 67.88)	59.02 (54.26 to 67.25)	.330
Left ventricular global longitudinal strain, %	−25.00 (−25.90 to (−21.95)	−22.40 (−26.35 to (−21.60)	.762
Cardiac adipose tissue (n = 15)			
Mean epicardial lipid content, mm^2^	1032.70 (894.23 to 1482.90)	929.37 (736.12 to 1317.15)	.022
Mean paracardial lipid content, mm^2^	1261.61 (1146.25 to 1831.17)	1295.30 (896.82 to 1755.58)	.639
Intramyocardial lipid content, %	0.52 (0.29 to 0.86)	0.32 (0.27 to 0.52)	.670

Abbreviations: BSA, body surface area; EDV, end diastolic volume; EF, ejection fraction; ESV, end systolic volume; HR, heart rate.

Intramyocardial lipid fraction (%) is calculated with the formula: (lipid peak area/(lipid peak area + water peak area)) × 100. Cardiac morphology and function parameters are normalized to BSA.

Left ventricle myocardial mass, end-diastolic, and end-systolic ventricular volume did not change at follow-up compared to baseline. No differences in parameters of systolic left ventricular function were observed.

EAT accumulation at baseline was associated with older age (*r* = 0.518; *P* = .048) and free serum cortisol concentrations (*r* = 0.596; *P* = .028). No correlations between changes in EAT and parameters of hypothalamus-pituitary-adrenal axis activity were observed.

All parameters on cardiac function and morphology, as well as cardiac fat stores at baseline and follow-up, are reported in Table 3. No relevant differences between patients with unilateral and bilateral disease were observed.

### Biochemical cardiovascular risk factors

Data of lipidomics analysis, BCAAs, and RAAS activity at baseline and follow-up are reported in detail in Table 4.

**Table 4:** Descriptive comparison of cardiovascular outcome parameters at baseline and follow-up (median; Q1-Q3)

Biochemical cardiovascular risk factors			
Lipidomics (n = 13) Total concentrations	Baseline	Follow-up	*P*
Triglycerides, mg/dL	128.25 (120.65-212.25)	144.59 (113.76-187.92)	.735
Cholesterol mg/dL	176.65 (162.56-185.99)	152.73 (148.80-181.88)	.244
Apo-A1, mg/dL	151.89 (132.63-155.37)	140.72 (127.95-147.00)	.497
Apo-A2, mg/dL	30.51 (27.62-34.44)	29.36 (27.16-32.86)	.273
total Apo-B100, mg/dL	75.82 (70.44-87.51)	67.57 (60.29-76.62)	.168
Apo-B100/Apo-A1 ratio	0.60 (0.48-0.62)	0.49 (0.42-0.54)	.152
LDL-cholesterol/HDL cholesterol ratio	1.76 (1.35-2.14)	1.40 (1.14-1.57)	.168
Lipoproteins and Apolipopoteins			
total particle No., nmol/L	1378.69 (1280.78-1591.09)	1228.61 (1096.26-1393.13)	.168
VLDL particle No., nmol/L	181.13 (137.71-279.55)	204.28 (158.54-227.42)	.839
IDL particle No., nmol/L	76.48 (64.31-102.08)	77.27 (71.7-101.13)	.787
LDL particle No., nmol/L	1065.33 (857.16-1362.99)	868.56 (750.26-1066.25)	.244
LDL-1 particle No., nmol/L	181.42 (159.95-237.95)	182.03 (160.9-207.22)	.497
LDL-2 particle No., nmol/L	123.60 (93.46-144.09)	77.96 (26.78-156.13)	.003
LDL-3 particle No., nmol/L	142.44 (113.57-155.29)	92.12 (51.15-125.50)	.216
LDL-4 particle No., nmol/L	158.77 (109.42-225.48)	138.67 (91.56-199.48)	.542
LDL-5 particle No., nmol/L	193.79 (138.05-233.43)	162.17 (128.79-210.81)	.340
LDL-6 particle No., nmol/L	277.47 (244.41-309.35)	262.5 (230.07-311.79)	.893
HDL Apo-A1, mg/dL	151.06 (130.12-156.75)	139.68 (125.34-144.57)	.497
HDL Apo-A2, mg/dL	31.76 (28.20-34.55)	29.31 (27.47-33.67)	.244
HDL-1 Apo-A1, mg/dL	14.72 (7.60-18.45)	14.88 (10.86-24.51)	.108
HDL-2 Apo-A1, mg/dL	16.34 (15.12-18.41)	15.74 (14.97-19.83)	.414
HDL-3 Apo-A1, mg/dL	26.74 (25.07-28.85)	24.33 (23.74-29.08)	.787
HDL-4 Apo-A1, mg/dL	82.16 (72.57-91.70)	79.15 (72.62-87.76)	.057
HDL-1 Apo-A2, mg/dL	1.42 (1.06-2.09)	1.37 (1.13-2.25)	.110
HDL-2 Apo-A2, mg/dL	2.49 (2.31-2.95)	2.63 (1.9-3.71)	.340
HDL-3 Apo-A2, mg/dL	6.05 (5.12-6.41)	5.68 (4.89-6.50)	.685
HDL-4 Apo-A2, mg/dL	20.94 (18.82-24.14)	19.49 (17.69-23.64)	.048
VLDL Apo-B100, mg/dL	9.96 (7.57-15.37)	11.23 (8.72-12.51)	.839
IDL Apo-B100, mg/dL	4.21 (3.54-5.61)	4.25 (3.94-5.56)	.787
LDL Apo-B100, mg/dL	58.59 (47.14-74.96)	47.77 (41.26-58.64)	.244
LDL-1 Apo-B100, mg/dL	9.98 (8.8-13.09)	10.01 (8.85-11.40)	.497
LDL-2 Apo-B100, mg/dL	6.80 (5.14-7.92)	4.29 (1.47-8.59)	.003
LDL-3 Apo-B100, mg/dL	7.83 (6.25-8.54)	5.07 (2.81-6.90)	.216
LDL-4 Apo-B100, mg/dL	8.73 (6.02-12.40)	7.63 (5.04-10.97)	.542
LDL-5 Apo-B100, mg/dL	10.66 (7.59-12.84)	8.92 (7.08-11.59)	.340
LDL-6 Apo-B100, mg/dL	15.26 (13.44-17.01)	14.44 (12.65-17.15)	.893
Triple A activity (n = 14)			
Angiotensin II (1-8), pmol/L	72.46 (27.73-95.27)	48.29 (34.69-94.16)	.855
Angiotensin I (1-10), pmol/L	24.45 (10.87-58.16)	21.75 (12.49-50.48)	.855
Aldosterone, pmol/L	105.09 (78.79-152.47)	75.40 (66.66-121.53)	.296
Aldosterone to angiotensin II ratio	1.56 (0.87-2.82)	1.21 (0.98-1.92)	.042
PRA-S, pmol/L	106.97 (38.55-226.42)	83.54 (49.88-182.68)	.761
ACE-S	3.26 (2.03-3.91)	2.98 (2.15-3.82)	.670
Branched chain amino acids (n = 14)			
Isoleucine, µmol/L	58.90 (53.71-70.28)	68.20 (54.05-79.05)	.173
Leucine, µmol/L	141.70 (127.25-157.20)	139.50 (118.27-176.98)	.903
Valine, µmol/L	312.55 (268.20-328.90)	289.05 (267.78-354.10)	.626
Sum: isoleucin,leucin and valine, µmol/L	509.64 (455.02-559.47)	484.85 (440.65-613.62)	≥.999

Abbreviations: ACE-S, angiotensin II/angiotensin I; APO, apolipoprotein; HDL, high-density lipoprotein; IDL, intermediate-density lipoprotein; LDL, low-density lipoprotein; PRA-S, plasma renin activity surrogate (angiotensin I + angiotensin II); VLDL, very-low-density lipoprotein.

### Lipidomics

Lipidomics analysis was performed in 13 patients before and after treatment due to analytical limitations. No patient had changes in lipid-lowering medication during the study period. The overall effects of treatment on total lipoprotein concentrations, apolipoprotein concentrations, and lipoprotein composition are displayed in Fig. 3. We observed a statistically significant decrease of −37% (0.63-fold) of low-density lipoprotein (LDL)-2 concentrations, that is medium-to-large sized LDL, and most related lipid parameters (total cholesterol, free cholesterol, phospholipids) in this lipoprotein subclass. Additionally, high-density lipoprotein (HDL)-4 cholesterol decreased significantly by −7% (0.93-fold). All data are reported in detail in Table 4 and Supplementary Table S2 (26).

**Figure 3:**
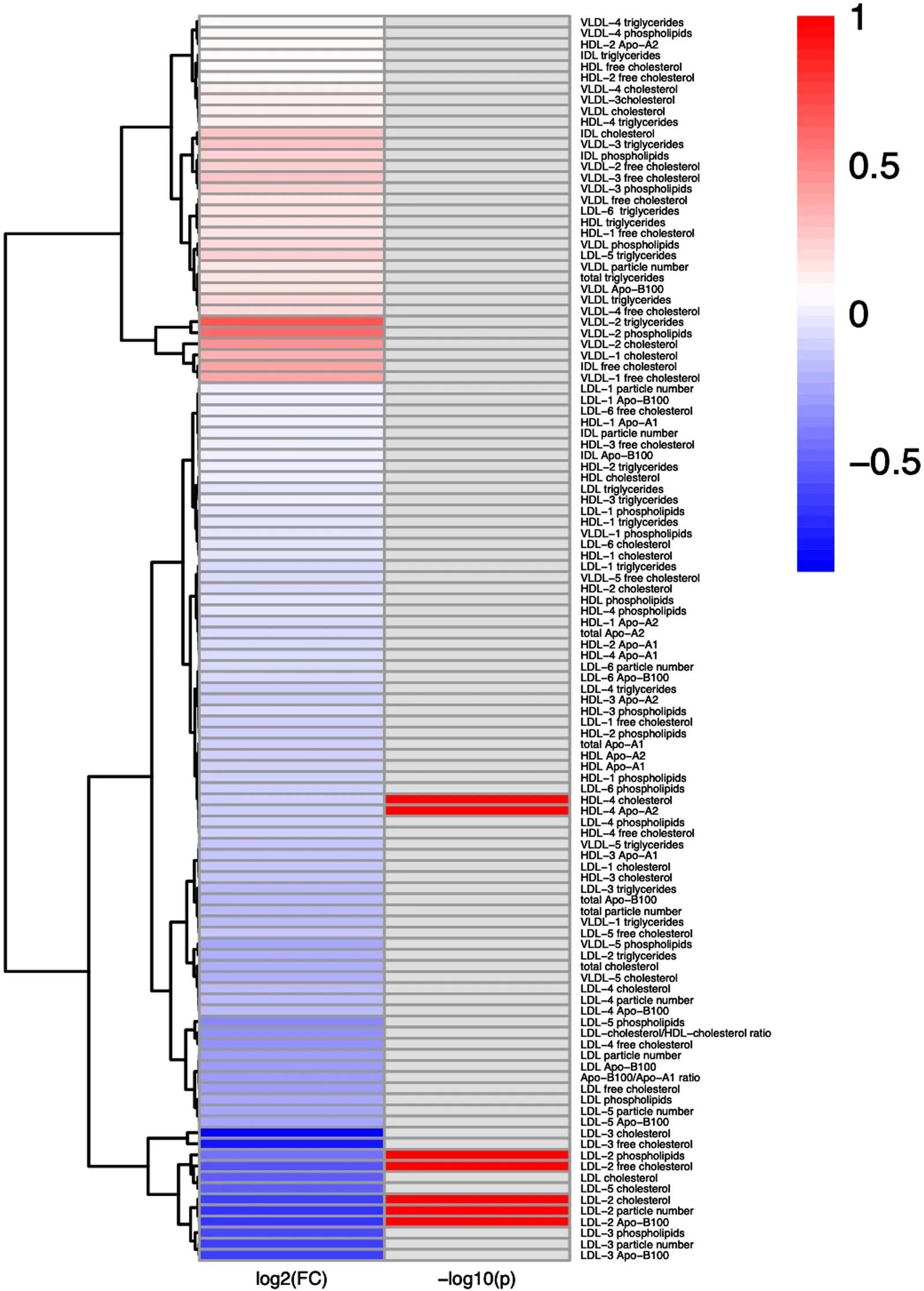
Lipidomics analysis comparing baseline and follow-up samples, expressed as log2 fold change (calculated: follow up/baseline value). Colors in the left column represent log2 fold change (follow-up/baseline), with shades of red indicating increased values at follow-up, white indicating no change, and shades of blue indicating decreased values at follow-up. The vertical bar on the right side indicates the corresponding log2 fold change scale. Statistically significant *P* values (*P* < .05) are indicated in red for easier readability in the second column.

### Branched chain amino acids

Comparisons of BCAA concentrations before and after treatment with metyrapone were performed in 14 patients and displayed no relevant change in concentrations of leucine, valine, and isoleucine (see Table 4).

### Renin-angiotensin-aldosterone system activity

Concentrations of aldosterone, angiotensin I, and angiotensin II were analyzed in 14 patients before and after treatment and are reported in Table 4. The AA2-ratio was significantly lower after treatment compared to baseline (Supplementary Fig. S1) (26).

### Therapeutic efficacy and safety

As previously reported (16), metyrapone treatment led to a statistically significant increase in ACTH (7 [4-13] vs 4 [4-6] pg/mL; *P* = .01) and 11-DOC (12.52 [7.69-15.22] vs 2.97 [2.17-4.30] ng/mL; *P* < .001), while morning cortisol levels were not significantly different (15.10 [13.10-18.50] vs 11.70 [9.98-14.38] µg/dL; *P* = .13) at follow-up compared to baseline. None of the patients reported any clinical signs of adrenal insufficiency. No serious adverse effects were observed.

## Discussion

In this prospective, proof-of-concept study, we combined state-of-the-art cardiac MRI and spectroscopy techniques with a standardized assessment of orthostatic arterial blood pressure regulation to evaluate the effects of evening doses of metyrapone in patients with MACS on cardiovascular risk factors. Treatment with metyrapone for 12 weeks significantly reduced blood pressure and EAT, features well known to be strongly involved in the pathogenesis of cardiomyopathy in patients with hypercortisolism. Our study highlights the adverse effects of MACS on the cardiovascular system that might be reversed by medical treatment with metyrapone.

Mean SBP and DBP in the supine position declined in the subgroup of patients without changes in antihypertensive medication. In line with our results, improvements in arterial hypertension were demonstrated in patients with MACS undergoing unilateral adrenalectomy (14, 27). In addition, a recent retrospective study of patients with MACS receiving evening doses of metyrapone reported decreases in SBP and DBP, respectively (28). The improvement in blood pressure following cortisol-lowering treatment in MACS might highlight its role as an important driver of secondary arterial hypertension. Moreover, changes in blood pressure might reflect a critical determinant of therapeutic efficacy of MACS treatment, which is easily assessable as a potential outcome parameter for treatment effectiveness in routine clinical care. No improvements in standing blood pressure might indicate that orthostatic regulation occurs independently due to baroreflex-receptor–mediated counterregulation, which is preserved after treatment in all patients.

The pathogenesis of arterial hypertension in a state of chronic hypercortisolism is complex. Among other mechanisms, changes in the activity of the RAAS together with direct stimulation of the mineralocorticoid receptor by high cortisol concentrations might be important (29, 30). We have previously reported a disease-specific profile of angiotensin metabolites in patients with ACTH-dependent CS (23), which cannot be observed here in patients with MACS. Following treatment with metyrapone, a decline in the AA2 ratio was observed. Whether this reflects a clinically significant downregulation of RAAS activity is difficult to interpret. Levels of 11-DOC, well known as a mineralocorticoid receptor agonist (31), increased with treatment and may be responsible for preventing a compensatory increase of RAAS activity despite a decrease in blood pressure. However, no statistically significant association between 11-DOC levels and components of the RAAS could be found in correlation analysis.

Another important finding of our study is that EAT is significantly lower after treatment with metyrapone, highlighting direct and reversible hypercortisolism-induced effects (8). Due to spatial proximity with the myocardium, epicardial fat is responsible for the paracrine or vasocrine release of proinflammatory peptides, which promote cardiac fibrosis and subsequently impair cardiac function. Moreover, EAT accumulation is involved in the development of coronary artery disease, atrial fibrillation, and heart failure (32).

So far, enhanced epicardial fat accumulation has been described in patients with CS and MACS (7-9). Additionally, a dose-dependent increase in EAT was reported in patients with long-term steroid intake (33). Successful treatment of hypercortisolism in patients with endogenous CS resulted in a prompt reduction in epicardial fat, but not paracardial fat (8). This is in line with our observations in patients with MACS. Paracardial fat might be affected rather indirectly by changes in body composition than directly by cortisol-lowering therapy, as it corresponds to a more “classic” visceral adipose tissue compartment (34), However, as reported previously, no changes in body weight or body composition were observed in our cohort (16), probably because of the short treatment duration.

No differences in intramyocardial fat were observed, which is again comparable to patients with CS (8).

With regard to cardiac function and morphology, we did not observe any differences following medical therapy. In a recent echocardiography-based study, left ventricular volume was smaller and interventricular septal thickness was more pronounced in patients with MACS compared to a group of age-, body mass index (BMI)-, and sex-matched controls (6). Longer treatment duration might be necessary to observe subclinical changes in parameters of cardiac geometry and function in patients with MACS, given the paucity of cortisol excess at baseline.

Hypercortisolism is associated with an increased prevalence of dyslipidemia, driven by hepatic synthesis and export of very-LDL (VLDL) and LDL, which is further worsened by cortisol-induced insulin resistance (35). Improvements in lipid parameters after adrenalectomy were found in small cohorts of patients with MACS (36), whereas surgery did not improve dyslipidemia according to another metanalysis (37). Of note, metabolic changes in a state of chronic hypercortisolism might drive a proatherogenic profile characterized by elevated concentrations of small LDL particles (38). In our study, by applying state-of-the art NMR spectroscopy, modest treatment-induced changes in lipid profiles could be identified (see Fig. 3). These were mainly characterized by a decrease of large-to-medium sized LDL-2 total particle number accompanied by a decline of individual LDL2-lipids and could be explained by changes in lipoprotein lipase activity.

Finally, we did not observe any changes in BCAA concentrations following treatment. BCAAs are well established cardiovascular risk markers and are associated with increased mortality. Increased BCAA concentrations were recently described in patients with adrenal adenomas with and without hormonal activity (3), whereas other studies reported even lower levels of BCAAs in patients with different degrees of endogenous hypercortisolism (39).

The major limitation of our study is the missing control group; therefore, the results must be considered exploratory rather than confirmatory. In addition, due to the small sample size and the exploratory assessment of multiple end points, values with borderline statistical significance should be interpreted with caution, as there is a risk of false-positive results, limiting the generalizability of our findings. However, given the paucity of evidence on the effects of medical treatment of MACS on cardiovascular risk factors, the aim of this secondary analysis of our proof-of-concept trial was to identify novel therapeutic outcomes. Future placebo-controlled studies are needed to confirm the beneficial effects of medical treatment.

Concomitant antihypertensive therapy and dose adaptations were not standardized prospectively, which is an additional limitation that might have attenuated the statistical significance of treatment-induced changes with respect to blood pressure. Following initiation of metyrapone, 4 patients independently reduced their long-term antihypertensive medication because of low blood pressure values during the study period. Consecutively, a subgroup analysis excluding patients with changes in antihypertensive therapy was conducted. However, this resulted in a smaller sample size.

### Conclusion

Medical treatment with evening doses of metyrapone improved arterial blood pressure in patients with MACS. EAT decreased significantly with treatment, while no changes in cardiac function or morphology were observed using cardiac MRI. The results of our study might reflect a causal role of hypercortisolism for the development of cardiovascular comorbidities in MACS that could be affected by chronotherapy with evening doses of metyrapone.

## Data Availability

Lipidomics and branched-chain amino acid data sets are accessible at the Mendeley Data repository (26). Remaining data are available from the corresponding author on reasonable request.

## Clinical trial information

EudraCT registration number 2022-000161-40 (registered August 6, 2022).

## Abbreviations

11-DOC: 11-deoxycortisol
^1^H-MRS: proton magnetic resonance spectroscopy
AA2 ratio: aldosterone to angiotensin II ratio
ACE-S: angiotensin II/angiotensin I
ACTH: adrenocorticotropin
BCAAs: branched-chain amino acids
BMI: body mass index
BSA: body surface area
CO: cardiac output
CS: Cushing syndrome
CYP3A4: cytochrome P450 3A4
DBP: diastolic blood pressure
DHEAS: dehydroepiandrosterone sulfate
EAT: epicardial adipose tissue
ED: end diastolic
EDV: end diastolic volume
EF: ejection fraction
ESV: end systolic volume
HDL: high-density lipoprotein
IDL: intermediate-density lipoprotein
LDH: lactate dehydrogenase
LDL: low-density lipoprotein
MACS: mild autonomous cortisol secretion
MRI: magnetic resonance imaging
PRA-S: plasma renin activity surrogate (angiotensin I + angiotensin II)
RAAS: renin-angiotensin-aldosterone system
SBP: systolic blood pressure
SV: stroke volume
VLDL: very-low-density lipoprotein
WC: waist circumference
